# Supplementary material for: Prognostic Significance of CSF-1R Expression in Early Invasive Breast Cancer
Source: Cancers (Basel). 2021 Nov 18;13(22):5769. doi: 10.3390/cancers13225769 (PMC8616299; doi:10.3390/cancers13225769)
Supplement: Supplementary file 1 [file cancers-13-05769-s001.zip › cancers-1417132-supplementary.pdf]

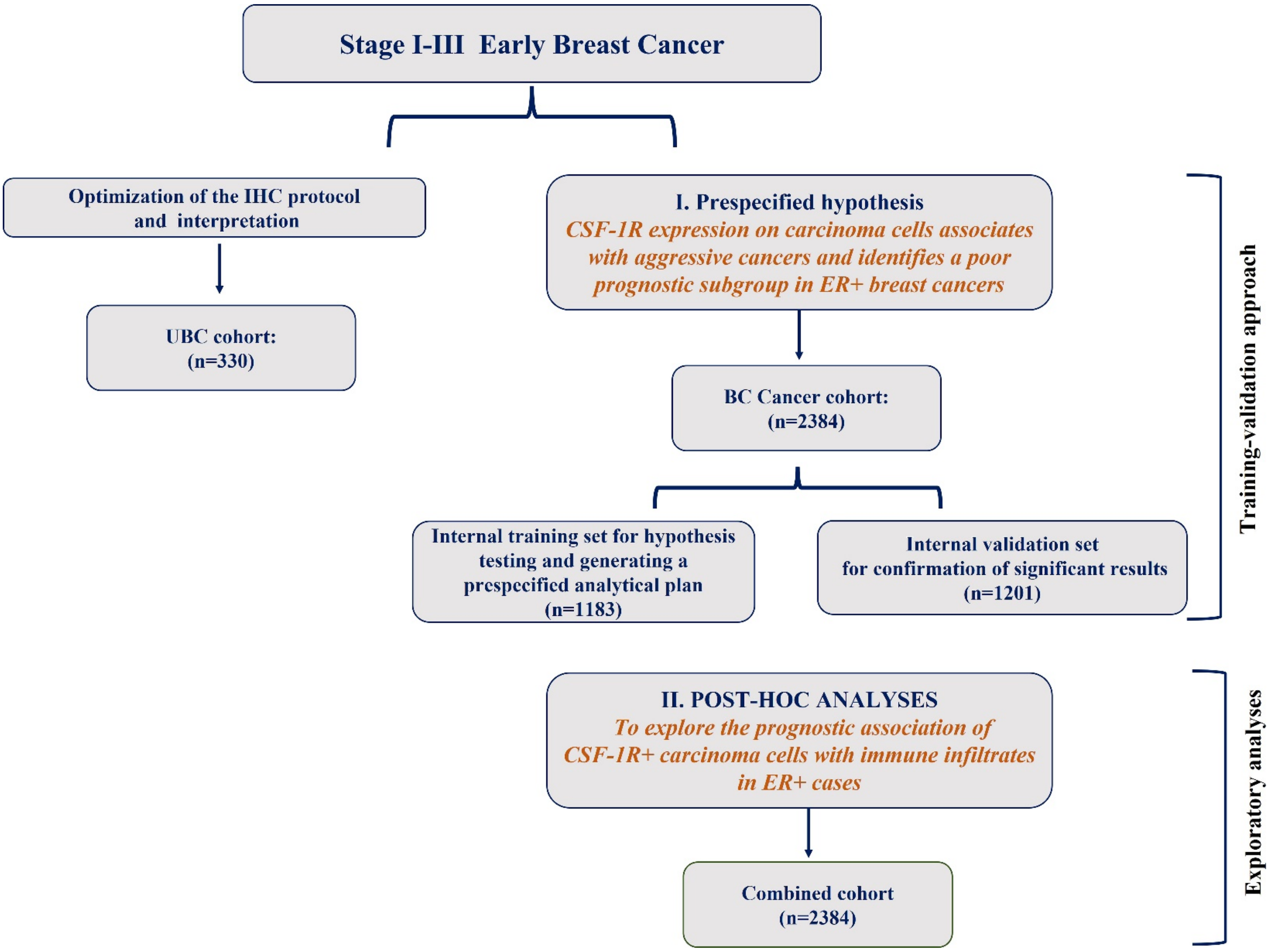

Figure S1. Flow-diagram illustrating the study design.

Table S1. Correlation of *CSF1R* mRNA with 37-gene tumor associated macrophage signature [1].

TAM signature genes	Purity-adjusted partial spearman's rho value	*p*-value
IRF8	0.686	3.75E-139
CCL2	0.396	1.06E-38
C1QC	0.746	1.97E-177
GBP5	0.486	2.73E-55
HCST	0.545	4.36E-78
LILRB4	0.719	6.06E-159
AIF1	0.790	4.25E-213
PSMB9	0.343	6.93E-29
GBP4	0.414	1.86E-42
GBP1	0.379	3.20E-35
HLA-DOA	0.725	5.10E-163
C1QA	0.664	1.50E-127
CCL4	0.526	7.25E-72
NCF1C	0.586	1.03E-92
LAP3	0.394	2.64E-38
TNFAIP3	0.501	2.21E-64
ITGB2	0.783	1.17E-206
LAIR1	0.792	8.87E-215

**TAM signature genes**	**Purity-adjusted partial spearman's rho value**	***p*-value**
FOLR2	0.528	2.31E-72
CD83	0.551	4.59E-80
SIGLEC1	0.603	1.45E-99
TCN2	0.450	1.33E-50
PLTP	0.466	8.80E-55
C1QB	0.703	7.61E-149
DOK2	0.608	1.99E-104
GIMAP6	0.581	6.40E-91
CD40	0.531	2.18E-73
CCL3	0.549	6.20E-53
CCL8	0.350	5.64E-30
FCN1	0.434	7.03E-47
CD4	0.838	8.07E-263
VAV1	0.642	2.00E-116
TLR7	0.750	1.78E-180
FGD2	0.742	1.29E-174
LST1	0.667	5.58E-129
VSIG4	0.730	1.79E-166
CLEC7A	0.620	1.72E-106

TAM, tumor associated macrophages. 37 genes represent the tumor associated macrophage signature described by Cassetta et al. (Reference # 24). (Spearman's rho: 0, no correlation; 0.1–0.3, weak; 0.4–0.6, moderate; 0.7–0.9, strong; 1, perfect; *p*-value < 0.05).

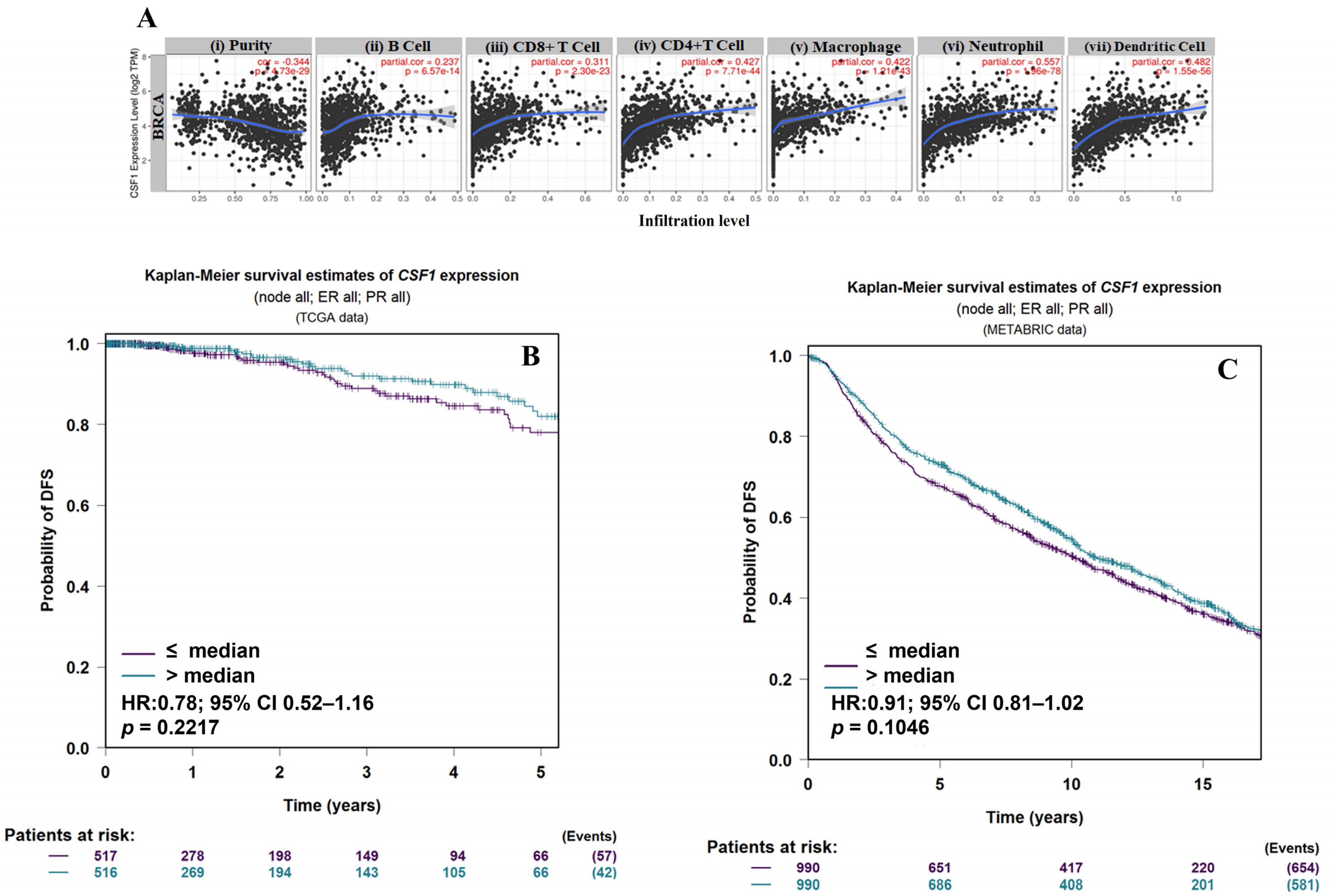
A
(i) Purity
(ii) B Cell
(iii) CD8+ T Cell
(iv) CD4+T Cell
(v) Macrophage
(vi) Neutrophil
(vii) Dendritic Cell
CSF1 Expression Level (log2 TPM)
BRCA
cor = -0.344
p = 4.73e-29
partial.cor = 0.237
p = 6.57e-14
partial.cor = 0.311
p = 2.30e-23
partial.cor = 0.427
p = 7.71e-44
partial.cor = 0.422
p = 1.21e-43
partial.cor = 0.557
p = 1.96e-78
partial.cor = 0.482
p = 1.55e-56
Infiltration level
Kaplan-Meier survival estimates of CSF1 expression
(node all; ER all; PR all)
(TCGA data)
B
Probability of DFS
≤ median
> median
HR:0.78; 95% CI 0.52–1.16
p = 0.2217
Time (years)
Patients at risk:
(Events)
517 278 198 149 94 66 (57)
516 269 194 143 105 66 (42)
Kaplan-Meier survival estimates of CSF1 expression
(node all; ER all; PR all)
(METABRIC data)
C
Probability of DFS
≤ median
> median
HR:0.91; 95% CI 0.81–1.02
p = 0.1046
Time (years)
Patients at risk:
(Events)
990 651 417 220 (654)
990 686 408 201 (581)

Figure S2. Correlation of *CSF1* mRNA expression with immune cell infiltrates and prognosis in breast cancer. TIMER2.0 web server was used to evaluate the correlation between *CSF1* mRNA expression and immune cell infiltrates. the left-most panel (i) shows *CSF1* mRNA expression against tumor purity (proportion of cancer cells in the sample), showing a significantly negative correlation. scatter plots (ii-vii) are showing the purity-adjusted partial spearman's rho value and statistical significance for positive correlation of *CSF1* mRNA with immune cell types (spearman's rho: 0, no correlation; 0.1-0.3, weak; 0.4-0.6, moderate; 0.7-0.9, strong; 1, perfect; p-value <0.05). five year Kaplan Meier curves showing an insignificant association of *CSF1* mRNA with disease free survival (DFS) in the TCGA (b) and METABRIC (c) invasive breast cancer cohorts. *p*-value and hazard ratios (HR) with corresponding 95% confidence intervals were estimated by the log-rank test. The survival curves for TCGA and METABRIC cohorts were generated using bc-GenExMiner v4.7.

Training cohort: correlation CSF-1R+ carcinoma cells with clinicopathological features, immune biomarkers and prognosis

The optimal scoring, and positivity thresholds were first finalized on the training cohort (n=1183). Of these, 23.6% demonstrated high expression (≥10%) with a significant positive association with age <50 years, high proliferation index (Ki67 ≥14%), negative expression of ER and progesterone receptor, positive expression of HER2 and non-luminal breast cancer subtypes (supplementary Table S2). In addition, CSF-1R+ carcinoma cells show a significant correlation with intraepithelial lymphocytes expressing PD-1, TIM3, LAG3, with PD-L1+ carcinoma cells and CSF-1R+ M2 macrophages (supplementary Table 3). Cases exhibiting high expression of CSF-1R by carcinoma cells are associated with significantly adverse breast cancer specific survival in the full training set (supplementary Figure 2A) and in ER positive cases (supplementary Figure 2C-D). In contrast, CSF-1R+ macrophages did not show any significant prognostic associations in the training cohort (supplementary Figure 2B). Cases with high CSF-1R+ carcinoma cells were associated with significantly greater hazard of breast cancer specific death in multivariate analysis, independent of the standard clinicopathological features (supplementary Table S4).

Table S2. BC Cancer series training set: correlation of CSF-1R+ carcinoma cells with clinicopathological features.

Clinicopathological variables	CSF-1R expression on carcinoma cells		*p*-value
	Low (<10%) 904 (76.4)	High (>10%) 279 (23.6)	
**Age at diagnosis**			
< 50	259 (28.7)	105 (37.6)	0.004*
≥ 50	645 (71.3)	174 (62.4)	
**Tumor size (cm)**			
≤ 2	482 (53.6)	131 (47.3)	0.07
> 2	418 (46.4)	146 (52.7)	
**Tumor grade**			
1 & 2	53 (6.2)	9 (3.3)	0.08
3	808 (93.8)	261 (96.7)	
**Axillary lymph node status**			
Negative	510 (56.5)	150 (54)	0.45
Positive	392 (43.5)	128 (46)	
**Lymphovascular invasion**			
Negative	475 (55.2)	143 (53.4)	0.60
Positive	386 (44.8)	125 (46.6)	
**ER expression**			
Negative	196 (21.7)	131 (47)	<0.001*
Positive	706 (78.3)	148 (53)	
**Progesterone receptor expression**			
Negative	345 (40.5)	176 (64.7)	<0.001*
Positive	506 (59.5)	96 (35.3)	
**HER2 overexpression/amplification**			<0.001*
Negative			

Positive	802 (90.9)	202 (73.5)	
	80 (9.1)	73 (26.5)	
**Ki-67 proliferation index**			
<14%	486 (58.4)	108 (40.3)	<0.001*
≥14%	346 (41.6)	160 (59.7)	
**Breast cancer subtypes (IHC)**			
Luminal NOS	42 (4.6)	3 (1.1)	
Luminal A	416 (46)	68 (24.4)	
Luminal B/HER2-/Ki67+	224 (24.8)	54 (19.4)	
Luminal / HER2+	47 (5.2)	26 (9.3)	<0.001*
HER2+	32 (3.5)	45 (16.1)	
Basal	69 (7.6)	47 (16.8)	
Additional basal by TNP	43 (4.8)	30 (10.8)	
Unassignable	31 (3.4)	6 (2.2)	

* Denotes differences between low and high CSF-1R groups that are significant at the Bonferroni-corrected p-value of <0.005 (0.05/10); ER, estrogen receptor; HER2, human epidermal growth factor receptor; TNP, triple negative phenotype.

Table S3. BC Cancer series training set: correlation of CSF-1R+ carcinoma cells with immune biomarkers.

Variables	CSF-1R+ carcinoma expression		p-value
	**Low (<10%)** **904 (76.4)**	**High (≥10%)** **279 (23.6)**	
**H&E sTIL count** (%)			
< 10	728 (86)	197 (74.1)	<0.001*
≥ 10	119 (14)	69 (25.9)	
**CD8 iTIL count**			
< 1	580 (67.2)	162 (60.4)	0.042
≥ 1	283 (32.8)	106 (39.6)	
**PD-1 iTIL count**			
< 1	803 (93)	230 (84.6)	<0.001*
≥ 1	60 (7.0)	42 (15.4)	
**PD-L1+ carcinoma cells (%)**			
0	789 (92.5)	223 (83.5)	<0.001*
≥ 1	64 (7.5)	44 (16.5)	
**FOXP3 iTIL count**			
< 2	577 (66.9)	157 (58.4)	0.010
≥ 2	285 (33.1)	112 (41.6)	
**TIM3 iTIL count**			
< 1	796 (91.1)	225 (82.1)	<0.001*
≥ 1	78 (8.9)	49 (17.9)	
**LAG3 iTIL count**			
< 1	786 (90.6)	225 (81.6)	<0.001*
≥ 1	82 (9.4)	50 (18.2)	
**CSF-1R macrophages**			
<2	544 (63.6)	117 (42.4)	<0.001*
≥2	312 (36.4)	159 (57.6)	
**CD163+ M2 macrophages**			
<2	332 (41.7)	75 (28)	<0.001*
≥2	465 (58.3)	193 (72)	

H&E, hematoxylin and eosin stained; iTILs, intraepithelial tumor infiltrating lymphocytes; sTILs, stromal tumor infiltrating lymphocytes; PD-1/L1, programmed cell death protein-1 /ligand 1; FOXP3, forkhead box P3; TIM3, T-cell immunoglobulin domain and mucin domain 3; LAG3, lymphocyte activation gene 3 protein; CSF-1R, colony stimulating factor-1 receptor.

BC Cancer cohort: training set
(*n*=1183)

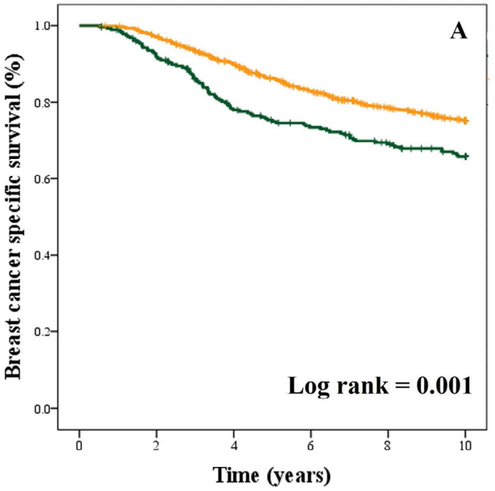

CSF-1R+ carcinoma cells < 10% (reference)
CSF-1R+ carcinoma cells ≥ 10% (HR 1.53, 95% CI 1.20–1.96)

BC Cancer cohort: training set
(*n*=1216)

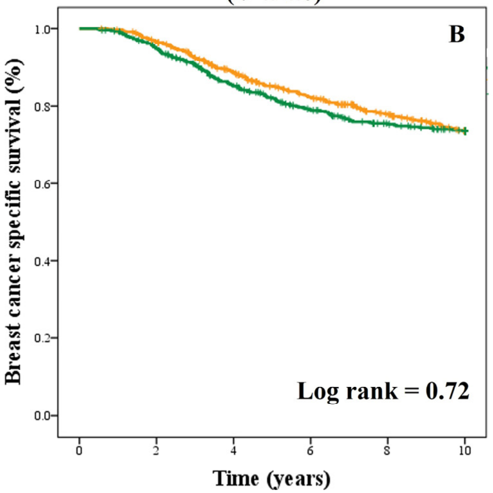

CSF-1R+ tumor associated macrophages < 2 (reference)
CSF-1R+ tumor associated macrophages (HR 1.04, 95% CI 0.83–1.31)

BC Cancer cohort: training set
ER positive subgroup (*n*=854)

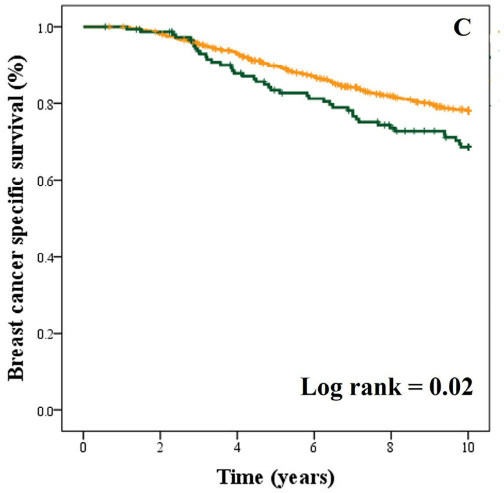

CSF-1R+ carcinoma cells <10% (reference)
CSF-1R+ carcinoma cells ≥ 10% (HR 1.51, 95% CI 1.07–2.13)

BC Cancer cohort: training set
ER negative subgroup (*n*=327)

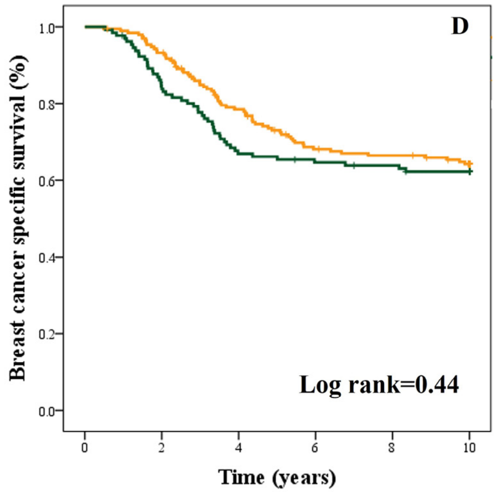

CSF-1R+ carcinoma cells < 10% (reference)
CSF-1R+ carcinoma cells ≥ 10% (HR 1.16, 95% CI 0.80–1.67)

Figure S3. Kaplan Meier survival curves showing breast cancer specific survival in the training set of BC Cancer series stratified by CSF-1R+ carcinoma cells (A) and CSF-1R+ tumor associated macrophages (B). Prespecified subgroup analysis for CSF-1R expressing carcinoma cells in ER positive cases (C) and ER negative cases (D). .

Table S4. Multivariate analysis for CSF-1R+ carcinoma cells in the training set of BC Cancer cohort.

Co-variates in the model	Breast Cancer Specific Survival	
	Adjusted HR (95% CI)	*p*-value
**Age at diagnosis (years)**		
<50	1	0.53
≥50	0.92 (0.72–1.83)	
**Tumor size (cm)**		
≤2	1	<0.001
>2	1.85 (1.43–2.4)	
**Tumor grade**		
1 & 2	1	<0.001
3	2.13-1.64–2.77)	
**Axillary LN status**		
Negative	1	<0.001
Positive	2.47 (1.86–3.29)	
**LVI**		
Negative	1	0.11
Positive	1.26 (0.95–1.66)	
**CSF-1R+ carcinoma cells**		
Low (<10%)	1	0.02
High (≥10%	1.38 (1.07–1.79)	

LN, lymph node; LVI, lymphovascular invasion; CSF-1R, colony stimulating factor-1 receptor.

References

1. Cassetta L, Fragkogianni S, Sims AH, et al. Human Tumor-Associated Macrophage and Monocyte Transcriptional Landscapes Reveal Cancer-Specific Reprogramming, Biomarkers, and Therapeutic Targets. *Cancer Cell.* 2019;35(4):588-602 e510.
